# Functional Near-Infrared Spectroscopy to Probe State- and Trait-Like Conditions in Chronic Tinnitus: A Proof-of-Principle Study

**DOI:** 10.1155/2014/894203

**Published:** 2014-11-16

**Authors:** Martin Schecklmann, Anette Giani, Sara Tupak, Berthold Langguth, Vincent Raab, Thomas Polak, Csanád Várallyay, Wilma Harnisch, Martin J. Herrmann, Andreas J. Fallgatter

**Affiliations:** ^1^Department of Psychiatry and Psychotherapy, University of Regensburg, Universitätsstraße 84, 93053 Regensburg, Germany; ^2^Max Planck Institute for Biological Cybernetics, 72076 Tübingen, Germany; ^3^Institute of Medical Psychology and Systems Neuroscience, University of Münster, 48149 Münster, Germany; ^4^Department of Psychiatry, Psychosomatics and Psychotherapy, University of Würzburg, 97080 Würzburg, Germany; ^5^Department of Neurosurgery, University of Würzburg, 97080 Würzburg, Germany; ^6^Department of Oto-Rhino-Laryngology, Plastic, Aesthetic and Reconstructive Head and Neck Surgery, University of Würzburg, 97080 Würzburg, Germany; ^7^Department of Psychiatry and Psychotherapy, University of Tübingen, 72076 Tübingen, Germany

## Abstract

*Objective*. Several neuroscience tools showed the involvement of auditory cortex in chronic tinnitus. In this proof-of-principle study we probed the capability of functional near-infrared spectroscopy (fNIRS) for the measurement of brain oxygenation in auditory cortex in dependence from chronic tinnitus and from intervention with transcranial magnetic stimulation. *Methods*. Twenty-three patients received continuous theta burst stimulation over the left primary auditory cortex in a randomized sham-controlled neuronavigated trial (verum = 12; placebo = 11). Before and after treatment, sound-evoked brain oxygenation in temporal areas was measured with fNIRS. Brain oxygenation was measured once in healthy controls (*n* = 12). *Results*. Sound-evoked activity in right temporal areas was increased in the patients in contrast to healthy controls. Left-sided temporal activity under the stimulated area changed over the course of the trial; high baseline oxygenation was reduced and vice versa. *Conclusions*. By demonstrating that rTMS interacts with auditory evoked brain activity, our results confirm earlier electrophysiological findings and indicate the sensitivity of fNIRS for detecting rTMS induced changes in brain activity. Moreover, our findings of trait- and state-related oxygenation changes indicate the potential of fNIRS for the investigation of tinnitus pathophysiology and treatment response.

## 1. Introduction

Chronic tinnitus is a highly prevalent condition (5–15%) [1] with no available effective therapy to cure this condition, that is, to turn off the tinnitus. Today, it is evident that chronic tinnitus is related to excitatory-inhibitory dysbalance of neural activity with increased spontaneous activity along the auditory pathway [2, 3]. Subsequent tonotopic reorganisation in the auditory cortex was also detected in animal models of tinnitus [4]. In humans, divergent findings are evident [5, 6].

In humans, increased activity in the auditory cortex is discussed as neuronal correlate of tinnitus [7, 8]. Early positron-emission tomography (PET) studies showed hints for a left-lateralization of activity of the primary auditory cortex in tinnitus [9]. In most cases longitudinal designs with modulation of the perception of tinnitus by lidocaine injection or somatic manoeuvres were used. A recent meta-analysis affirmed increased activity in the left primary auditory cortex and showed also increased activity in bilateral auditory cortex [10]. A more recent study contrasting patients and controls [11] challenged this concept by providing evidence that left-lateralization is independent from tinnitus.

In contrast to PET which measures brain metabolism over several minutes, functional magnetic resonance imaging (fMRI) is able to measure resting state and sound-evoked activity. Resting state studies mainly showed increased coherent activity of auditory areas [12, 13] with occasional reports of no changes between patients and controls [14]. Sound-evoked studies showed increased activity in auditory cortex [15] or asymmetric activity in correspondence to the lateralization of the tinnitus [16]. There are also reports of no changes in auditory cortex [17, 18]. One recent study showed decreased connectivity between the inferior colliculi and the auditory cortices [17]. In conclusion, tinnitus seems to be associated with increased activity and connectivity of auditory cortex.

Also studies using electroencephalography (EEG) and magnetoencephalography (MEG) showed changes in auditory evoked potentials and resting state power and connectivity. Most of these studies investigated the association of tinnitus characteristics with physiological markers of brain activity in correlational designs [19].

Because of the pathophysiological involvement of the auditory cortex in chronic tinnitus, the noninvasive brain stimulation method repetitive transcranial magnetic stimulation (rTMS) was introduced as a possible treatment option [20–22]. Despite initial positive treatment effects, it turned out over the course of the last 11 years that effect sizes of clinical trials using rTMS for treating tinnitus are moderate, interindividual variability is high, and there is still no effective cure [23]. Based on demographic and clinical data, there is no clear indicator or biomarker for rTMS treatment response or treatment effects in chronic tinnitus [24]. Neurophysiological and neuroimaging data might represent a more objective and reliable index of the treatment response than subjective ratings. EEG [25] and MEG [26] resting state and auditory evoked fMRI and EEG [27] measures were associated with rTMS treatment effects. Thus, the evaluation of rTMS efficacy by neuroimaging constitutes the next step in tinnitus research [28]. This strategy may increase the efficacy of rTMS in tinnitus by explaining the interindividual variability enabling a more individualized treatment.

Functional near-infrared spectroscopy (fNIRS) is a neuroimaging method which enables the measurement of calottenear brain activity by measuring blood oxygenation level dependent signals based on principles of optical topography [29–31]. Despite moderate spatial and depth resolution, the big advantages of fNIRS over fMRI are the silent and upright measurement setting, mobility, handiness, little movement artifacts, and high temporal resolution [30]. Particularly in tinnitus, it is of high relevance to have as little machine noise as possible due to interaction effects with auditory cortical activity.

The aim of the present study was to probe the capability of fNIRS as a potential indicator for tinnitus-related activity in the auditory cortex. For this aim we contrasted brain oxygenation of patients with chronic tinnitus and healthy controls and before and after treatment with rTMS over the left auditory cortex. We expect that sound-evoked activity is increased in chronic tinnitus and that hyperactivity can be decreased by cTBS. As the left temporal hemisphere was stimulated we expect effects to take primarily place on this hemisphere.

## 2. Material and Methods

### 2.1. Samples and Procedures

The study has been approved by the local ethics committee of Wuerzburg (Germany) and has been performed according to the declarations of Helsinki. Twenty-three patients with chronic tinnitus were enrolled in the study after having given written informed consent. Patients with acute or chronic inflammation of the middle ear, Menière disease, sudden idiopathic hearing loss, or fluctuating hearing were excluded. Otologic assessment included micro-otoscopy, pure-tone audiometry, tympanometry, and stapedius reflex measurement to verify normal middle ear function. Mean hearing loss in dB HL was defined as the average pure-tone hearing threshold of both ears at 0.125, 0.250, 0.500, 1, 2, 3, 4, 6, and 8 kHz. Patients with a history of seizures, a suspected diagnosis of organic brain damage, and pregnancy as well as patients with cardiac pacemakers, mobile metal implants, or implanted medication pumps were excluded.

Clinical effects and study design will be published in detail separately. In sum, we conducted a randomised sham-controlled pilot trial (verum: *n* = 12; sham: *n* = 11) using continuous theta burst stimulation of the left Heschl's gyrus targeted with anatomical neuronavigation. Stimulation was performed twice (2 × 600 pulses with a short break) on five days per week, starting on Monday, with an intermediate break over the weekend between the first and last five days of treatment. Stimulation intensity was set at 30% of the maximum stimulator output. fNIRS measurements were performed before (2 weeks before stimulation) and after treatment (on the day of the last treatment). Healthy controls (*n* = 12) were measured once with fNIRS. Sample characteristics are provided in Table 1.

### 2.2. Sound-Evoked Activity

We used one block and one event-related design for measurement of oxygenation changes induced by acoustic stimulation. “Comité Consultatif International Télégraphique et Téléphonique” (CCITT) speech noise was presented binaurally by means of insert earphones (E-A-RTONE3A, Aero Company, USA). The tip of the earphones was placed into the auditory canal, guaranteeing an exact adjustment of the sound intensity. Intensity level was set to 70 dB SPL. For the block design, participants listened to 12 blocks of CCITT noise. Each block lasted 20 s and was followed by a 20 s resting period. For the event-related design, stimuli were presented 40 times with a variable interstimulus interval of 12–14 s for 1.75 s.

### 2.3. Measurement of Brain Activity

**Figure 1:**
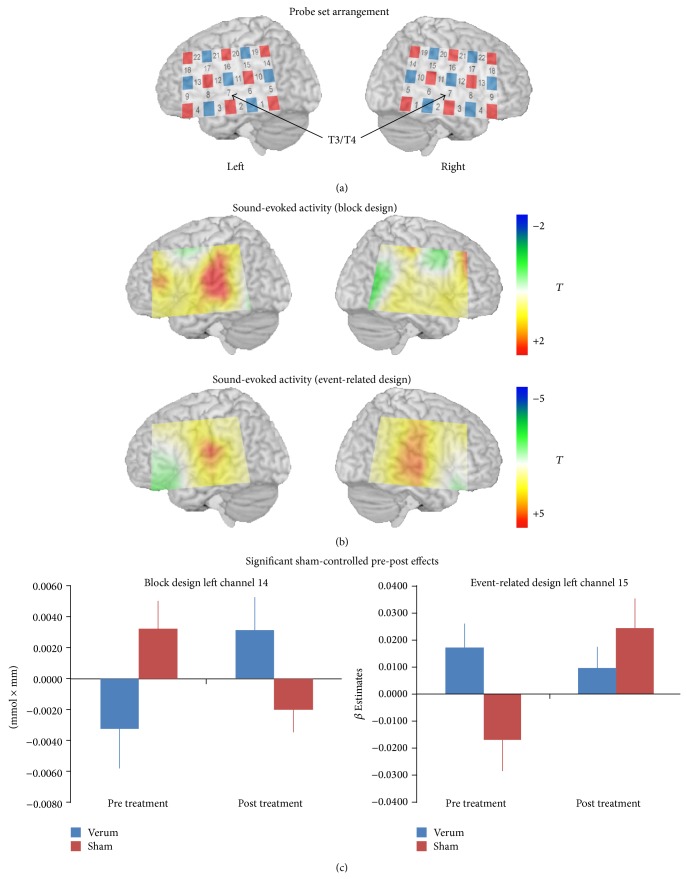
Localisation of light emitters (red squares) and detectors (blue squares) and measurement channels (numbers) for near-infrared spectroscopy (a). Sound-evoked activity for all subjects at baseline (*n* = 35; tinnitus patients and healthy controls) as indicated by brain oxygenation (b). Channels with significant group by time interaction effects (c).

Functional near-infrared spectroscopy (fNIRS) has been shown to have high validity [32] and reliability [33–35] and is suited to measure sound evoked activity in the auditory cortex [36]. For the fNIRS measurement, we used a continuous multichannel wave system (ETG4000 Optical Topography System; Hitachi Medical Co., Japan) working with two different wavelengths (695 ± 20 nm and 830 ± 20 nm) and a time resolution of 10 Hz to measure relative changes of absorbed near-infrared light. These changes are transformed into concentration changes of oxygenated (O_2_Hb) and deoxygenated haemoglobin (HHb) as indicators for brain activity by means of a modified Beer-Lambert law [37]. The unit is mmol × mm; that is, changes of chromophore concentration depend on the path length of the near-infrared light. We used two identical rectangular probe sets (plastic panels) of optodes (light emitters and detectors) for each side of the head. One probe set consisted of 8 light emitters and 7 detectors with an interoptode distance of 3 cm. A measuring point of activation (channel) was defined as the region between one emitter and one detector. Thus, one probe set consisted of 22 channels and covered an area of 6 × 12 cm on the scalp. The panels were fastened to the head by elastic straps. The probe sets were placed on the head with regard to the relevant standard positions of the international 10–20 system for EEG electrode placement [38, 39]. The channel over the middle inferior optode was placed over T3/T4 with vertical orientation in direction to C3/C4. The arrangement of the probe set is shown in the top of Figure 1.

### 2.4. Data Analysis and Statistics

Before statistical analysis of fNIRS data, the high frequency portion of the signal was removed by calculating a moving average with a time window of 5 seconds. During normal physiological activity neurovascular decoupling is accompanied in local increases in O_2_Hb and simultaneous decreases in HHb resulting in negative correlations of both chromophores. Correlations that tend to be positive or equal to zero may indicate noise caused by motion or extracerebral or systemic hemodynamic activity unrelated to the experimental condition [37]. A correlation based signal improvement algorithm was used to filter out spikes and to improve signal quality based on the assumed negative correlation between O_2_Hb and HHb [40]. The applied correction results in one single parameter of brain oxygenation which is a mathematical combination of O_2_Hb and HHb.

For the block design measurement, slow drifts in the measurement were excluded by the use of a linear fitting resulting from calculation of the mean of the 10 s right before the auditory stimulation and the mean of the interval between the 10th and 20th second after the stimulation. A mean trajectory for each condition was calculated by averaging the 12 repetitions. For statistical analyses, we used the mean amplitude of oxygenation during the stimulation period. For the event-related design, slow drifts in the signal were excluded by the use of a high pass filter with six discrete cosine basis functions. We used the general linear model approach according to fMRI and fNIRS literature [41–43]. Association of channels and brain areas were deducted from the work of Okamoto and colleagues [39], which interrelates EEG positions and fNIRS probe sets. Analyses were done by home-made scripts on MatLab (The MathWorks Inc., USA).

For statistical analyses of imaging data we used two-sided Student *t*-tests and analyses of variance (ANOVAs) for each channel of each probe set. Firstly, for reasons of plausibility we contrasted the activity (amplitude for the block and beta values for the event-related design) of each channel against zero to test for region-specific activity of auditory areas. These basal brain activations were indicated by two-sided Student *t*-tests against zero and by according T-maps interpolated for all channels over the whole probe set (Figure 1). Secondly, we analysed overall group differences for the baseline measurements and contrasted the group of patients and the group of controls with unpaired *t*-tests. Thirdly, to evaluate intervention specific pre-post effects we conducted ANOVAs with the independent factor group (verum versus sham) and the dependent factor time (pre versus post). Post hoc analyses were done for significant interactions by using *t*-tests. To take baseline differences into account, we repeated the ANOVAs with significant interaction effects by including the baseline values as covariate (ANCOVAs). Due to 22 calculations per probe set we used a Bonferroni correction by using the Dubey/Armitage-Parmar alpha boundary taking into account spatial correlation among the fNIRS channels [44–47]. Based on the baseline measurements the mean correlation coefficient was 0.539 resulting in an adjusted alpha level of 0.0123. Thus, we just report *P* values below 1.23%. For post hoc tests and the ANCOVAs we abstained from using Bonferroni correction. All statistical analyses were performed with SPSS 20.0.0.1 (SPSS Inc., USA).

## 3. Results

For all measured subjects (verum and sham patients and control group) both designs showed activity over auditory areas. Topographies of brain oxygenation in the form of T-maps are shown in Figure 1. Statistical values were higher for the event-related design in contrast to the block-design experiment as indicated by the color bar ranges of Figure 1. In line, only for the event-related design we found significant activity (left hemisphere: channel 11: *P* < 0.001; channel 16: *P* = 0.010; right hemisphere: channel 2: *P* = 0.009; channel 6: *P* = 0.005; channel 7: *P* < 0.001; channel 11: *P* < 0.001; channel 16: *P* < 0.001; channel 20: *P* = 0.007). Nonsignificant activity can be seen only over the left auditory areas for the block design.

In the next step we analysed overall group differences for the baseline measurements. *t*-tests between patients and controls showed group differences in the right auditory cortex as indicated by significant differences in right channels 2 (*P* < 0.001) and 7 (*P* = 0.006) for the block and in the right channel 12 for the event-related design (*P* = 0.004). The significant findings of the block design were related to auditory areas; patients had higher oxygenation changes in contrast to controls. The significant channel of the event-related design was related to frontal areas showing decreased activity in the patient group. To sum up the baseline findings, oxygenation in the right hemisphere differed between patients and controls; that is, patients showed higher auditory oxygenation during the block design and more focused activation during the event-related design as mirrored by decreased activation in frontal areas. In the left hemisphere there were no clear differences between patient groups and the control group.

Pre-post changes showed no significant group × time interaction effects on the right hemisphere—neither for the block nor for the event-related design. For the block design, channel 14 showed a significant interaction effect (*P* = 0.005) (Figure 1). During baseline, the sham group had higher oxygenation in contrast to the verum group (*P* = 0.057). At the last treatment visit, this relation was reversed (*P* = 0.061). The verum group displayed higher oxygenation than the sham group. Oxygenation decreased in the sham group over the course of the trial (*P* = 0.059) and increased for the verum group (*P* = 0.039). The effect was stable by controlling for baseline differences using an ANCOVA with baseline values as covariate (*P* = 0.041).

In the event-related design, left channel 15 showed a significant interaction effect in the opposite direction as compared to the effect in the block design (*P* = 0.005). The verum group had higher oxygenation during the baseline in contrast to the sham group (*P* = 0.025) with no oxygenation differences during the last treatment visit (*P* = 0.294). Oxygenation increased in the sham group over the course of the trial (*P* = 0.012) with no changes for the verum group (*P* = 0.476). The effect disappeared by controlling for baseline differences using an ANCOVA with baseline values as covariate (*P* = 0.135). To sum up the pre-post effects, the activity of the right hemisphere was not sensitive to specific treatment effects. For the left hemisphere, increased oxygenation during baseline decreased over the course of the treatment and lowered oxygenation during the baseline increased over the course of the treatment with reversed patterns between the verum and sham group and between the block and event-related design. Findings of the ANCOVA indicate that these interaction effects are highly dependent from the baseline values.

## 4. Discussion

In line with a former study [36], we were able to measure sound-evoked activity in temporal areas with fNIRS. A finding of the present study was a reversed pattern in brain oxygenation between the block and event-related design. For the right hemisphere, activity differences between patients and controls were mirrored by increased activity for the block design and rather focused activity in the event-related design in the patients. For the left hemisphere, oxygenation was changing during treatment with reversed patterns for the verum and the sham group. For the block design, the sham group showed reductions from an increased activity level over the course of treatment; the verum group showed the opposite pattern. This interaction was reversed in the event-related design. Differences between the block and event related design have already been proposed as a potential explanation for the limited reliability in fMRI research [48]. Our findings suggest that short sounds within the range of 1-2 s in contrast to long-lasting sound stimulation within the range of several seconds might lead to differences in neural activation. Differences in brain activation dependent on stimulus duration seem to be reasonable. In contrast to a short salient sound, the same sound becomes irrelevant if presented for a longer time and is presumably inhibited via top-down mechanisms. This might be in accordance with models of prepulse inhibition and sensory gating for which a preceding stimulus decreases the novelty of the incoming information [49, 50]. The sensitivity of fNIRS for top-down mechanisms in the auditory domain has been documented in an earlier fNIRS study which detected an influence of the emotional valence on sound induced temporal oxygenation [36]. Whether the observed differences between the event-related design and the block design are a general phenomenon or specific for tinnitus has to be elucidated by further studies.

A further notion is that our data did not clearly indicate a favourable sound-evoked design to measure activity specific for tinnitus and/or specific for rTMS interventions over the auditory cortex. The event-related design showed higher baseline *T*-values and clear bilateral activity in contrast to the block design with smaller *T*-values and only left-sided activity for the whole group (Figure 1). On the other hand rTMS related changes in the block design seem to be more stable for baseline differences in pre-post designs.

One advantage of fNIRS is the possibility to easily combine it with noninvasive brain stimulation to measure TMS induced activity changes [51, 52]. In the present trial rTMS and fNIRS were performed successively because of the interest in the neurobiological change induced by a therapeutic intervention. The present findings showed trait related increases of brain oxygenation in the right auditory areas as indicated by increased activity in the block design and more focused activity in the event-related design. Increased activity in patients is in line with several studies using sound-evoked fMRI [16, 53] or tinnitus-related alterations in oxygenation or metabolic brain activity [10]. This increased activity is considered to represent the tinnitus percept or at least one aspect of the tinnitus percept [54, 55]. The quiet measurement setting of fNIRS affirms the fMRI findings. The latter method might have been confounded by scanner background noise due to the radio waves and cooling pumps, which limits the interpretation of fMRI findings in tinnitus [56].

Our results further suggest that left auditory cortex oxygenation may reflect state-like effects. Based on the baseline level (block design: sham > verum; event-related design: verum > sham) the intervention led to a decrease in the group with increased baseline activity and vice versa (block design: verum > sham; event-related design: sham > verum). The importance of these pre-post effects is highlighted by the ANCOVAs. The interpretation of these findings is challenging because of the difference in baseline activity between these two groups. Thus it is difficult to disentangle, to which extent the inverse changes during treatment depend on the stimulation procedure (verum versus sham) or on the difference in baseline measurements. Nevertheless our findings are in accordance with a recent fMRI study in tinnitus showing the same neural pattern [27]. Our findings of treatment related changes in temporal blood flow are also comparable to findings of temporoparietal rTMS for the treatment of auditory hallucinations in schizophrenia. In twelve medication-resistant patients, PET revealed a reduction in symptoms and left temporoparietal metabolism after low frequency of the corresponding area [57]. In another study cerebral blood flow (arterial spin labeling) in the left superior temporal gyrus predicted treatment response [58]. Open questions for future work are the meaning of negative baseline values during baseline and if the pretreatment differences are due to differences in samples. These might be resolved by more rigorous characterization of the samples, by increasing the sample size, by repeated baseline measurements, and by correlation of baseline values with tinnitus characteristics.

Similar like in sound evoked fMRI or PET studies, we have to acknowledge that it still has to be elucidated, how the observed alterations of sound evoked activity in tinnitus relate to its neuronal correlates. Alterations in sound evoked activity can only provide an indirect hint for the neuronal changes underlying tinnitus.

Altogether, even if we cannot provide final explanations for all aspects of our fNIRS results, the data confirm the usefulness of fNIRS for neuroimaging of auditory function. Future studies including the same design in control subjects should shed even more light to trait- and state-like effects of rTMS on auditory cortex activity in tinnitus. Open questions in tinnitus research might be evaluable with this method such as association of tinnitus characteristics (e.g., laterality) with temporal activity [11, 16] or combination with EEG to verify source localisation of power changes [59, 60]. Also for the investigation of other areas such as the orbitofrontal cortex fNIRS has a higher suitability because of susceptibility artifacts in fMRI for these areas [61]. Generally, other fields of phantom perception such as acoustic hallucinations might be investigable with fNIRS [62].

## 5. Conclusions

The present proof-of-principle study showed that fNIRS is able to measure brain oxygenation changes of the auditory cortices in relation to tinnitus. Furthermore, the combination of noninvasive brain stimulation and neuroimaging offers the detailed investigation of trait- and state-related biomarkers for tinnitus. As fNIRS measures calotte-near brain oxygenation changes which means rather secondary than primary auditory cortex and as rTMS findings also indicate secondary and temporoparietal areas as potential treatment targets [63, 64], the question arises if fNIRS might serve as indicator for functional neuronavigation which is the stimulation of the nonprimary auditory cortical areas with sound-evoked related activity.

## Figures and Tables

**Table 1:** Sample characteristics.

	Verum patient group [*n* = 12]	Sham patient group [*n* = 11]	Healthy control group [*n* = 12]	Statistics for group contrasts
Age [years]	48.2 ± 10.7	46.5 ± 11.5	43.6 ± 15.0	*F* = 0.407; df = 2, 32; *P* = 0.669
Sex [female/male]	5/7	4/7	3/9	*χ* ^2^ = 0.770; df = 2; *P* = 0.680
Mean hearing loss [dB HL]	20.5 ± 11.0	21.4 ± 6.7	—	*T* = 0.223; df = 16; *P* = 0.826
Tinnitus duration [months]	68.9 ± 61.4	96.8 ± 120.4	—	*T* = 0.702; df = 20; *P* = 0.491
Tinnitus laterality [unilateral/not unilateral]	2/10	2/8	—	*χ* ^2^ = 0.041; df = 1; *P* = 0.840
Tinnitus questionnaire [0–84]	39.4 ± 11.8	46.3 ± 15.9	—	*T* = 1.181; df = 21; *P* = 0.251
Tinnitus handicap inventory [0–100]	42.3 ± 17.5	50.2 ± 18.7	—	*T* = 1.039; df = 21; *P* = 0.311
Beck depression inventory II [0–63]	11.6 ± 8.8	7.0 ± 7.2	—	*T* = 1.356; df = 21; *P* = 0.190
